# Evaluation of the dental educational environment in upper Egypt using the DREEM tool: a cross-sectional study

**DOI:** 10.1186/s12909-025-08340-y

**Published:** 2025-12-13

**Authors:** Shereen Shaaban Mustafa, Wael Felefel, Shereen Hafez Ibrahim

**Affiliations:** 1 Department of Pediatric Dentistry and Dental Public Health Faculty of Dentistry, Beni-Suef University, Beni-Suef, Egypt; 2 Department of Parasitology, Faculty of Veterinary Medicine, Matrouh University, Marsa Matruh, Egypt; 3 Department of Conservative Dentistry, Faculty of Dentistry, Cairo University, Cairo, Egypt

**Keywords:** Educational environment, DREEM, Dental education, Medical education, Student perception, Learning environment

## Abstract

**Background:**

Assessment of the educational environment (EE) encompasses an evaluation of all elements that influence students’ learning experiences to enhance the quality of dental education programs. The Dundee Ready Education Environment Measure (DREEM) is a reliable tool that evaluates perceptions of undergraduate students on their EE through five subscales. Accordingly, the current study aimed to assess the dental EE in Upper Egypt using the DREEM tool.

**Methods:**

This study was conducted throughout the academic year (2024–2025) at the Faculty of Dentistry, Beni-Suef University (FDBSU). An electronic questionnaire was distributed among undergraduate students. It included the English and Arabic versions of the DREEM questionnaire. Independent *t*-tests and one-way ANOVA (*F*-test) have been used to examine differences in the total DREEM scores and the subscales among different academic years and academic phases. The Cronbach’s alpha test was used to assess the reliability of the questionnaire.

**Results:**

From a total of 495 approached students, 235 students responded to the questionnaire (response rate of 47.5%). The total DREEM score was 128.59 ± 25.4, indicating a more positive than negative environment. The Cronbach’s Alpha test value was 0.94. There were no statistically significant differences total DREEM score between students across all demographic variables, including gender, academic phase, nationality, and residence location. There were significant differences across different academic years in Students’ Perception of Learning (SPL) (*P* = 0.043) and Students’ Academic Self-Perception (SAP) (*P* = 0.025), while other subscales showed no significant differences across different academic years.

**Conclusions:**

Perceptions of students regarding the EE reveal equality and quality that are positive across genders, nationalities, or preclinical and clinical phases, providing all students with the necessary tools and opportunities to attain their full potential. Customized support may be necessary across various academic years in two subscales, SPL and SAP. Improvements in any of these elements will positively impact all aspects of the EE.

**Supplementary Information:**

The online version contains supplementary material available at 10.1186/s12909-025-08340-y.

## Introduction

 Over the last few decades, significant changes in dental education took place globally and in the Arab region, particularly in Egypt. This was crucial to ensure these changes meet international standards by employing integrated strategies and technologies that enhance the learning experience and prepare future healthcare professionals. A recent study stated that Egypt, as a part of the WHO Eastern Mediterranean region, includes forty-three universities that offer both undergraduate and postgraduate dental programs to Egyptian and international students [1]. This reflects the significant role of dental education in Egypt since the beginning of history that affects the Egyptian population, along with the populations of nearby countries in the Middle East and Africa [1–4].

Providing high-quality education and training to undergraduate dental students is essential for their success during their academic years and later in their careers [5]. In medical education, the EE has a significant role in preparing well-qualified doctors as it impacts students’ competencies, morals, and ethics. It can also reflect the standards of the medical institute from which they received their education [6].

**Fig. 1:**
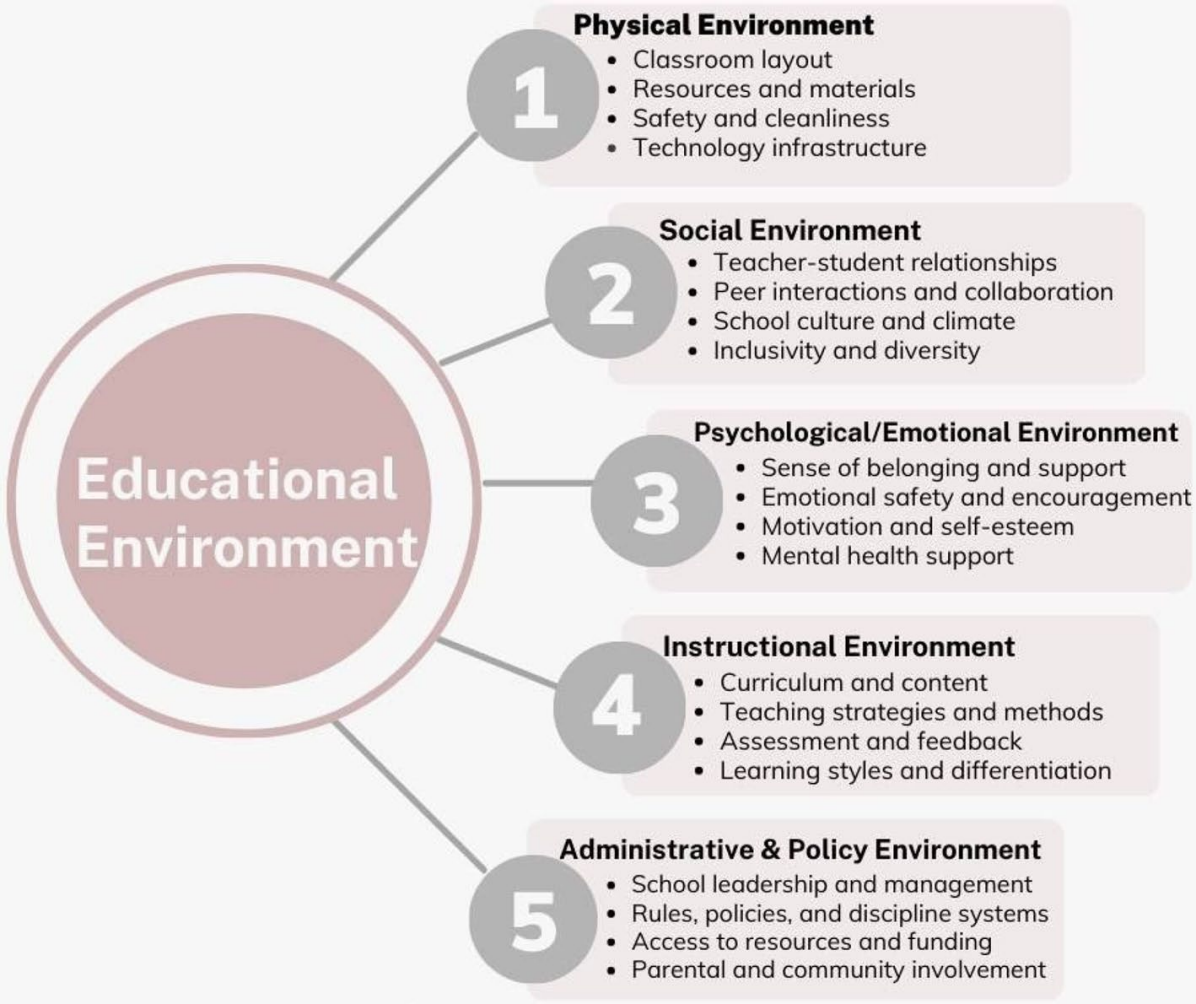
The components of the educational environment

In accordance with the American Medical Association, the educational environment includes the learners, the related stakeholders, the purpose of learning, the settings, and all the administrative logistics associated with education [7]. Accordingly, the institutional culture, infrastructure, physical space, curriculum design, and the interactions of students with their colleagues, staff members, and college officials have a significant impact on shaping the EE as presented in (Fig. 1) [6, 8, 9].

The ideal dental EE should prepare students to properly attain and use the required academic knowledge to become competent in all the required clinical and interpersonal aspects of their careers. Additionally, it should respect their physical and psychological well-being and help in the advancement of their self-confidence, culture, and ethics [5]. Regarding the physical aspects of the EE represented in the lecture halls, Classrooms, teaching facilities, laboratories, and clinics, should be properly designed to allow for safe and comfortable seating arrangements with minimum external disturbances [5, 10].

Over the past few decades, multiple tools have been developed to enable measuring and assessing the medical EE depending on perceptions of students toward their EE [11, 12]. Examples of the measuring tools include: the Dundee Ready Education Environment Measure (DREEM) [13], the Johns Hopkins Learning Environment Scale (JHLES) [14], and the Anatomy Education Environment Measurement Inventory (AEEMI) [15].

**Fig. 2:**
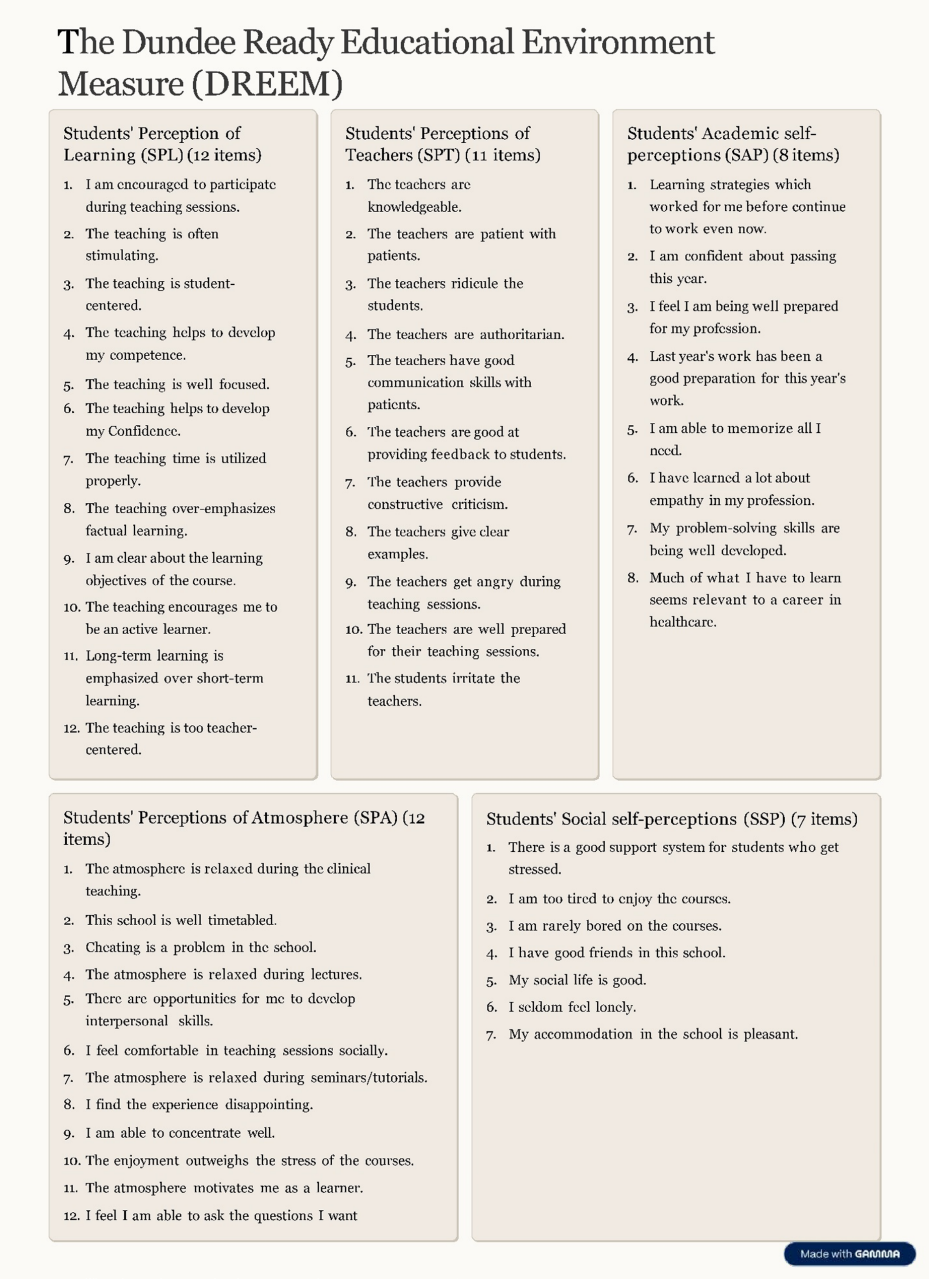
The Dundee Ready Educational Environment Measure (DREEM) subscales items

In 1997, Roff et al. developed the DREEM tool, and since then, it has become almost the most commonly used validated and reliable tool that evaluates the EE in medical, dental, and nursing schools [13–16]. DREEM as presented in (Fig. 2 ) is a questionnaire that includes 50-statement closed questions subdivided into 5 subscales as follows: Students’ perception of learning (12 items), Students’ perceptions of teachers (11 items), Students’ academic self-perceptions (8 items), Students’ perceptions of atmosphere (12 items), and Students’ social self-perceptions (7 items) 17]. These domains collectively reflect both cognitive and ethical aspects of education, assessing how effectively the EE supports knowledge acquisition while promoting respect, fairness, and student well-being. This comprehensive approach makes DREEM a valuable tool for identifying strengths and areas needing improvement in medical education ethically and academically, compare students among different academic years, and even compare different medical schools. Accordingly, DREEM became a valuable guide in strategic planning for curriculum modification, identification of educational priorities, and resource utilization in medical education [18, 19].

Multiple dental institutions have been established in Upper Egypt aimed to provide students with the necessary logistics and proximity to their residences. Although DREEM has been globally used for the assessment of students’ perception of their EE in multiple medical and dental schools [13, 20–24], no literature has been found to use the DREEM tool for the assessment of the dental EE in Egypt. Studying Upper Egypt using the DREEM questionnaire is academically important due to the unique educational challenges and cultural context of this region. Upper Egypt used tofaces disparities in healthcare education quality, resource limitations, and distinct sociocultural factors that may influence students’ learning experiences and ethical development. Assessing the educational environment in this area provides critical insights into how these factors affect student perceptions and outcomes, helping tailor interventions that promote fairness, respect, and effective education aligned with the national standards [3]. 

Given the foregoing background, the research questions raised in our study were: Is the DREEM questionnaire a reliable tool to evaluate the dental EE in Egypt? Will the dental EE be adequate in a governmental faculty of dentistry in Upper Egypt at different academic years? What possible gaps in the dental EE in the Egyptian universities do educators encounter from students’ perception?

The null hypothesises of this study are that the DREEM questionnaire might not be a reliable tool to evaluate the dental EE in Egypt, students’ perception of their dental EE will be equivalent among students at different academic years The aim of this cross-sectional study was to investigate the perceptions of students on their dental EE in Upper Egypt using the DREEM tool and provide recommendations aiming to contribute to the improvement of the dental EE in Upper Egypt.

## Methodology

### Study design and setting

The current cross-sectional study was conducted at the Faculty of Dentistry, Beni-Suef University during the academic year 2024–2025 between December 2024 and April 2025. Ethical approval was obtained from the Research Ethics Committee in the FDBSU, with approval number (# REC-FDBSU/05122024-02/MS). An electronic questionnaire was developed to be distributed among participants using Google Forms. It started with an electronic informed consent, denoting that voluntary submission of the questionnaire was considered as implied consent for participation.

### Participants

Based on a calculated sample size, a minimum number of 215 undergraduate students needed to be approached to participate in this study [25]. This was achieved by targeting all the undergraduate students enrolled at the FDBSU for the academic year 2024–2025 (*n* = 495) during multiple lectures and practical sessions. They all received a verbal explanation of the aim of the study, scientific terms mentioned in the questionnaire, and the impact of their free opinion on enhancing the quality of teaching and other aspects of their EE in the future. Afterward, they all received an email from the primary investigator through their official university email, including the link to the Google Form and a message clarifying that participation is non-compulsory and all responses are confidential and unidentified. The same content was sent to the students through their WhatsApp groups with the help of the students’ representative from each academic level. Students’ responses were electronically collected till they reached the determined sample size. Undergraduate students enrolled in other faculties, interns, and postgraduate students were not eligible for the current study.

### Sample size

The Raosoft sample size software has been used for sample size calculation based on the number of students in FDBSU (495 students) and the response rate (43.40%) from a study previously conducted in Saudi Arabia. This study assessed the perceptions of students toward the EE at the Faculty of Dentistry, King Abdulaziz University, using the same questionnaire consisting of 50 questions [25]. Under these conditions, with a precision of 5 and an α of 5%, the number of participants needed for the current study was 215. Convenience voluntary response sampling was utilized to recruit participants in this study [26, 27].

### Measures

**Fig. 3:**
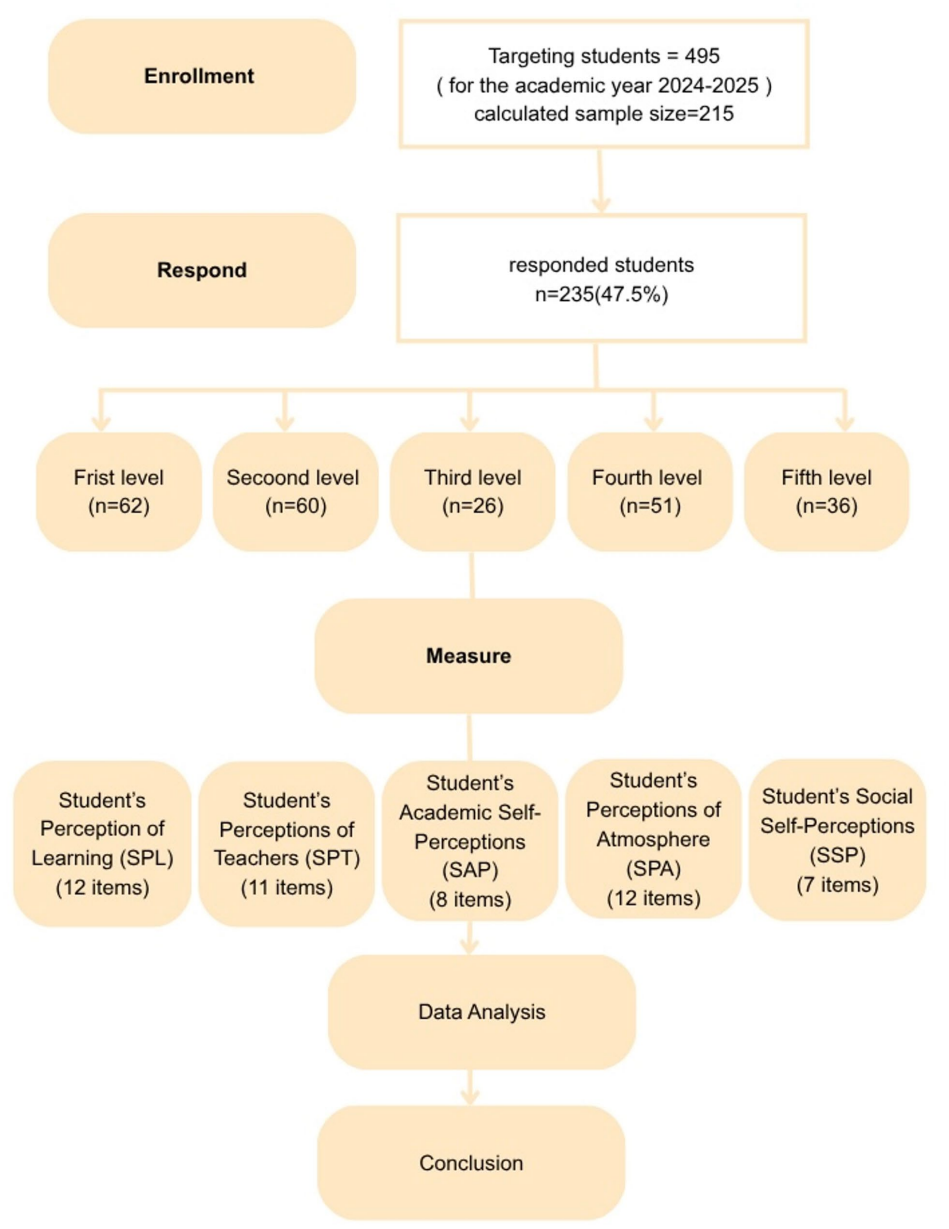
flow diagram summarizing sampling and data analysis steps

A survey consisting of two parts was developed to evaluate the dental EE in FDBSU. The first part included closed-ended questions aimed at gathering demographic data, including gender, nationality, residence location, and academic level of education. The second part included the English and Arabic versions of the valid pre-tested bilingual adaptation of DREEM questionnaire to ensure clarity of all items as each question was presented in both languages [17, 28]. The 50-statement closed questions were divided into five subscales as follows: Students’ Perception of Learning (SPL) (12 items), Students’ Perceptions of Teachers (SPT) (11 items), Students’ Academic Self-Perceptions (SAP) (8 items), Students’ Perceptions of Atmosphere (SPA) (12 items), and Students’ Social Self-Perceptions (SSP) (7 items) as described in Fig. 3 Each of the 50 statements was scored using a 5-point Likert scale as follows: ‘Strongly agree’ (4), ‘Agree’ (3), ‘Unsure’ (2), ‘Disagree’ (1), and ‘Strongly Disagree’ (0). The DREEM has a maximum score of 200, with 0–50 indicating a very poor EE, 51–100 indicating plenty of problems in the EE, 101–150 indicating more positive than negative EE, and 151–200 indicating an excellent EE. All items that included a reverse statement have been properly identified, and their scores were reversed during data entry and analysis as follows: 0 for ‘Strongly agree’, 1 for ‘Agree’, 2 for ‘Unsure’, 3 for ‘Disagree’, and 4 for ‘Strongly Disagree’ [28].

**Fig. 4:**
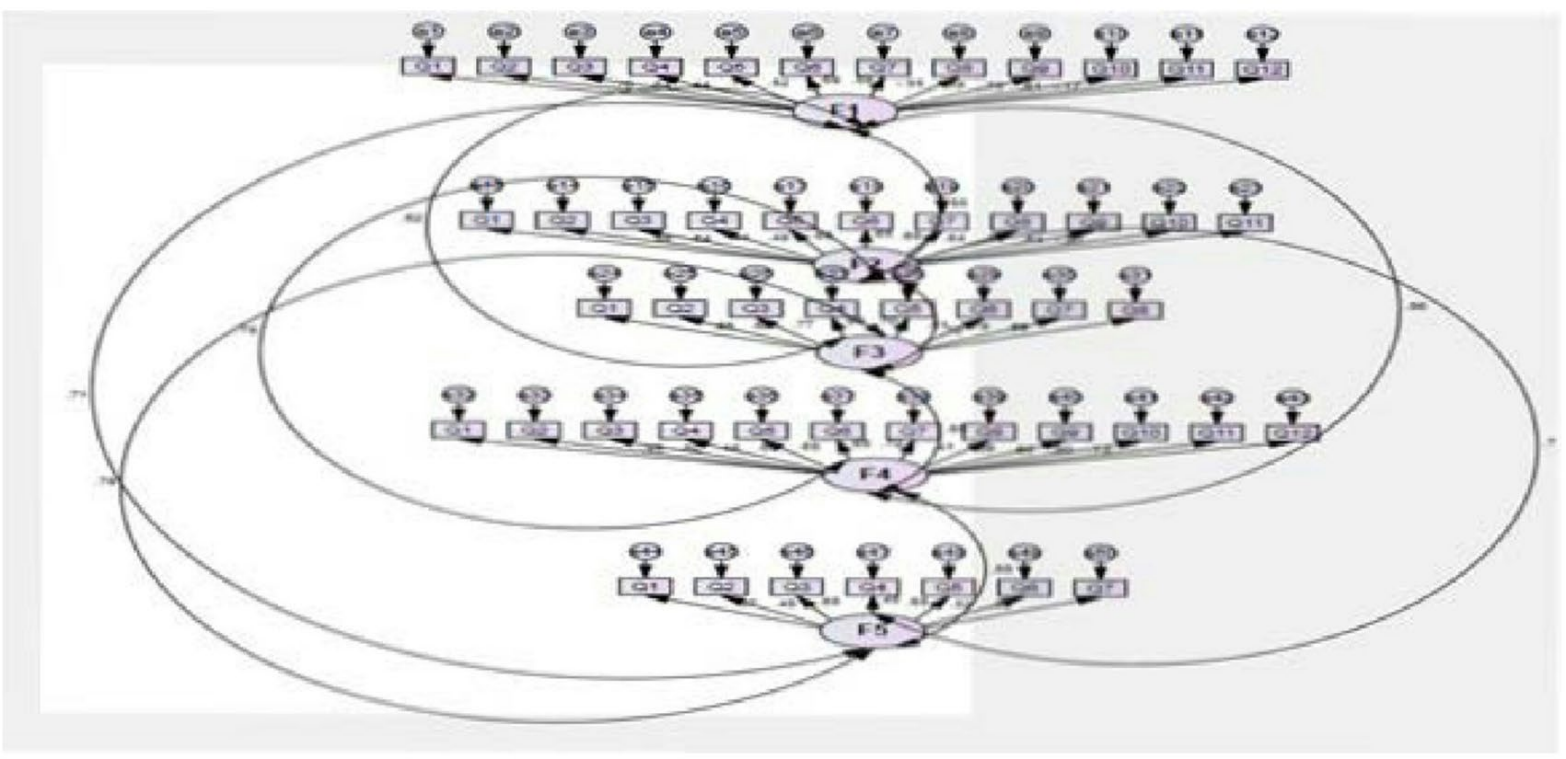
Confirmatory factor analysis was subsequently conducted using AMOS software version 22 to confirm the five-factor structure

The item’s mean score was interpreted as follows: 3.5 or higher indicates excellent educational aspects, 3.01–3.49 signifies positive educational qualities, 2.01–3.00 indicates room for improvement, and 2.00 or below points to problematic educational aspects [29] Fig. 4.

### Statistical method

Data was analyzed using SPSS version 22, with a significance level set at 0.05, all qualitative variables, were presented using frequency and percentage, followed by Z-test to compare between different proportions and the association between dental educational environment categories and academic years was examined using the Monte Carlo test (2-sided)., on other hand quantitative variables were expressed by mean ± standard deviation (x ± SD), followed by test of normality by using Kolmogorov–Smirnov test then independent t-tests and one-way ANOVA (F-test) were used to compare between different means. Then questionnaire analysis included reliability test by Cronbach’s alpha and exploratory factor analysis (EFA) by Kaiser–Meyer–Olkin (KMO) and, Bartlett’s test with rotation level 0.4 followed by confirmatory factor analysis was subsequently conducted using AMOS software version 22 to confirm the five-factor structure. Finally, correlations among the five subscales of the dental EE questionnaire were evaluated using Pearson’s correlation coefficient.

## Results

Table 1 showed that the total DREEM score was 128.59 ± 25.4. It also presented that the reliability of the questionnaire was excellent, as the Cronbach’s Alpha test value was 0.94.

Regarding data presented in the supplementary materials Table 5 Supp showed that all five dental EE questionnaire subscales were valid as the value of each was above 0.7, and the Kaiser-Meyer-Olkin KMO value was above 0.5. Bartlett’s test was significant, implying that the questionnaire could be divided into factors or domains (factorability) with variability in participants’ responses. Furthermore, by using principal axis factoring in Table 1, we reported that the 5 subscales of the questionnaire explained 43.094% of the dental educational environment. There was a strong correlation between each question and the total questionnaire score. None of the questions could be excluded as the communalities extraction values were above 0.1. AMOS analysis presented in (Fig. 3) revealed that the model was a significant fit, Chi-square = 2468.674, *P* < 0.001.

**Table 1:** The factor analysis, and reliability of the questionnaire in the current study

Subscale Items	mean ± SD	Cronbach’s Alpha	Kaiser-Meyer-Olkin	Bartlett’s Test	Rotation Sums of Squared Loadings total variance explained
Total DREEM score	128.59 ± 25.4	**0.94**	0.92	0.000	43.09%

### Extraction Method

Principal Axis Factoring. Regarding the demographic variables presented in Table 2, a total of 235 students responded to the questionnaire, with a 47.5% response rate. Participants included 105 males (44.7%) and 130 females (55.3%) with no significant difference in the response rate between genders. Participants from the preclinical phase represented 62.55% while those from the clinical phase (37.45%), showing a significant difference in the response rate between the preclinical and clinical phases (*P* = 0.0002). Both nationality and residence location showed significant differences in the response rate among participants (*P* = 0.00001). Additionally, there were no significant differences in the total questionnaire scores across all demographic variables.

**Table 2:** The basic demographic data of participants

Variables		Frequency	%	*P*	DREEM score	T/F	*P*
Gender	Male	105	44.7	0.10	130.18 ± 27.35	0.86^T^	0.34
	Female	130	55.3		127.30 ± 23.73		
Academic year of education	First year	62	26.4	0.11	127.06 ± 25.15	1.9^F^	0.11
	Second year	60	25.5		131.22 ± 25.71		
	Third year	26	11.1		123.65 ± 26.30		
	Fourth year	51	21.7		123.94 ± 25.06		
	Fifth year	36	15.3		136.97 ± 23.81		
Academic phase	pre-clinical	147	62.55	0.0002	128.73 ± 24.65	0.11	0.91
	Clinical	88	37.45		128.35 ± 26.74		
Nationality	Egyptian	220	93.6	0.00001	129.24 ± 24.67	1.50^T^	0.13
	non-Egyptian	15	6.4		119.07 ± 34.04		
Residence location	Beni-Suef	151	64.25	0.00001	127.70 ± 26.21	− .72^T^	0.47
	other governorates	84	36.75		130.19 ± 23.95		

-T, independent t test, F, One-way ANOVA test, Z test

Table 3 demonstrates that there is no statistically significant difference between the preclinical and clinical phases in the five subscales of the questionnaire.

**Table 3:** The mean of subscales according to the academic phase

questionnaire subscale	Academic phase	*N*	Mean ± SD	t	*P*
Students’ Perception of Learning (SPL)	pre-clinical	147	30.42 ± 5.96	− 0.51	0.61
	clinical	88	30.83 ± 5.83		
Students’ Perceptions of Teachers (SPT)	pre-clinical	147	30.2585 ± 5.33	-1.43	0.15
	clinical	88	31.2614 ± 4.98		
Students’ Academic Self-Perceptions (SAP)	pre-clinical	147	21.26 ± 5.31	− 0.26	0.8
	clinical	88	21.44 ± 5.45		
Students’ Perceptions of Atmosphere (SPA)	pre-clinical	147	30.04 ± 7.45	1.06	0.29
	clinical	88	28.88 ± 9.16		
Students’ Social Self-Perceptions (SSP)	pre-clinical	147	16.75 ± 5.10	1.15	0.25
	clinical	88	15.94 ± 5.35		

-T, independent t test

Results presented in Table 4 showed significant differences across different academic years in Students’ Perception of Learning (SPL) (*P* = 0.04) and Students’ Academic Self-Perception (SAP) (*P* = 0.03), while other subscales showed no significant differences across different academic years.

**Table 4:** The mean DREEM score for each subscale among different academic years

Questionnaire subscales	Academic years	N	Mean ± SD	One-way ANOVA	
				F	P
Students’ Perception of Learning (SPL)	First year	62	29.85 ± 6.11	**2.50**	**0.04**
	Second year	60	30.80 ± 5.87		
	Third year	26	30.12 ± 7.11		
	Fourth year	51	29.57 ± 5.53		
	Fifth year	36	33.19 ± 4.51		
	Total		30.57 ± 5.90		
Students’ Perceptions of Teachers (SPT)	First year	62	30.50 ± 5.03	**1.34**	**0.26**
	Second year	60	30.2667 ± 5.49		
	Third year	26	29.2692 ± 6.00		
	Fourth year	51	30.8431 ± 4.61		
	Fifth year	36	32.1667 ± 5.17		
	Total		30.6340 ± 5.22		
Students’ Academic Self-Perceptions (SAP)	First year	62	20.65 ± 5.59	**2.83**	**0.03**
	Second year	60	22.43 ± 5.33		
	Third year	26	19.38 ± 4.87		
	Fourth year	51	20.71 ± 5.46		
	Fifth year	36	22.94 ± 4.54		
	Total		21.33 ± 5.35		
Students’ Perceptions of Atmosphere (SPA)	First year	62	30.32 ± 7.73	**1.67**	**0.16**
	Second year	60	30.28 ± 7.79		
	Third year	26	27.85 ± 7.41		
	Fourth year	51	27.65 ± 9.23		
	Fifth year	36	31.28 ± 7.91		
	Total		29.60 ± 8.13		
Students’ Social Self-Perceptions (SSP)	First year	62	15.74 ± 5.48	**2.00**	**0.10**
	Second year	60	17.43 ± 4.99		
	Third year	26	17.04 ± 4.85		
	Fourth year	51	15.18 ± 5.12		
	Fifth year	36	17.39 ± 5.10		
	Total		16.45 ± 5.2		

One-way ANOVA test

The correlation between the 5 subscales of the dental EE questionnaire was presented in Table 5, showing that there was a significant positive intermediate to average correlation between the 5 subscales of the dental EE questionnaire.

**Table 5:** The correlation between the 5 subscales of the dental educational environment questionnaire

Subscales	Correlation coefficients	Students’ Perception of Learning (SPL)	Students’ Perceptions of Teachers (SPT)	Students’ Academic Self-Perceptions (SAP)	Students’ Perceptions of Atmosphere (SPA)	Students’ Social Self-Perceptions (SSP)
Students’ Perception of Learning (SPL)	Pearson Correlation	1				
	Sig. (2-tailed)					
	N	235				
Students’ Perceptions of Teachers (SPT)	Pearson Correlation	0.62^**^	1			
	Sig. (2-tailed)	0.000				
	N	235	235			
Students’ Academic Self-Perceptions (SAP)	Pearson Correlation	0.68^**^	0.57^**^	1		
	Sig. (2-tailed)	0.000	0.000			
	N	235	235	235		
Students’ Perceptions of Atmosphere (SPA)	Pearson Correlation	0.72^**^	0.66^**^	0.76^**^	1	
	Sig. (2-tailed)	0.000	0.000	0.000		
	N	235	235	235	235	
Students’ Social Self-Perceptions (SSP)	Pearson Correlation	0.53^**^	0.56^**^	0.64^**^	0.71^**^	1
	Sig. (2-tailed)	0.000	0.000	0.000	0.000	
	N	235	235	235	235	235
**. Correlation is significant at the 0.01 level (2 tailed)						

-Monte Carlo Sig. (2-sided)

Table 6 presents challenging items in the EE that had a mean value less than 2, indicating problems in the related elements of the EE.

**Table 6:** Challenging items in the education environment

Subscale	Item	Mean ± SD
Students’ Perception of Learning (SPL)	The teaching over-emphasizes factual learning.	0.91 **±** 0.8
	The teaching is too teacher-centered.	1.46 **±** 1.01
Students’ Perceptions of Teachers (SPT)	The students irritate the teachers.	1.86 **±** 1.23
Students’ Perceptions of Atmosphere (SPA)	The enjoyment outweighs the stress of the courses.	1.95 **±** 1.22
Students’ Social Self-Perceptions (SSP)	I am too tired to enjoy the courses	1.55 **±** 1.14
	I am rarely bored on the courses	1.95 **±** 1.14

## Discussion

In the last few decades, significant attention has been given to educational research to assess and improve the EE in dental education worldwide [30]. Systematic evaluation of the EE enables educators and administrators to identify strengths and weaknesses in the curriculum, teaching strategies, and student support services, which reflect in student satisfaction, motivation, and academic achievement [13, 31]. Fairness, social justice, respect for human dignity, and a dedication to giving everyone the resources, and chances they need to reach their full potential are the foundations of the ethical aspect of educational quality and equality. Quality education becomes a universal human right rather than a privilege as it goes beyond simply providing the same education to everyone (equality) to guaranteeing customized support to remove systematic disadvantages (equity) [17, 30, 31]. This could be manifested in terms of practical and policy aspects into 5 main pillars. Resource Allocation is the first pillar to solve inequalities between wealthy and low-income communities, policies for the fair distribution of funds, technology, and educational resources are informed by ethics. Inclusive Pedagogy and Curriculum as a second pillar where educators have an ethical duty to modify curriculum materials and teaching strategies to accommodate the varied requirements of every student, incorporating various viewpoints and guaranteeing cultural sensitivity. The third pillar was educator conduct described as honesty, accountability, empathy, and upholding appropriate professional boundaries are all stressed by professional ethics. Furthermore, educators influence students’ moral growth by acting as role models, and this was the fourth pillar. Whereas, the fifth pillar was addressing systemic bias, it is morally required of educational programs to confront and eradicate institutional biases that can sustain inequality, such as those seen in standardized testing or disciplinaryprocedures [17, 28, 30, 31]. Thus, in order to develop interventions that support equity, respect, and successful instruction that is in line with national standards, it is crucial to evaluate the educational environment in Upper Egypt in order to get insights into how these aspects affect student perceptions.

The current cross-sectional study is the first to investigate students’ perceptions of their dental EE in Upper Egypt however, other studies in medical education in Egypt were reported [32].

The DREEM tool has been used in this study as it is a universal, validated questionnaire that has effectively enabled researchers to assess the EE in numerous faculties around the globe [20, 21, 29, 33, 34]. It was developed in 1997 at the University of Dundee in Scotland by Roff et al. using a combination of qualitative and quantitative methods, including Grounded Theory and Delphi procedures, involving nearly 100 global health education experts and over 1,000 students from different countries. The tool was designed to be culturally independent and multi-dimensional to assess the EE in health professions education worldwide [17]. The questionnaire was distributed among undergraduate students using Google Forms. It was a user-friendly, cost-effective tool that enabled easy and widespread distribution of the questionnaire among students, with real-time monitoring of data collection and analysis [35]. Both the English and Arabic versions of the (DREEM) questionnaire had been used to ensure the clarity of English vocabulary and prevent miscomprehension, given that English is a second language for the students with diverse proficiency levels among them [28, 36].

The Faculty of Dentistry, Beni-Suef University (FDBSU), located in Beni-Suef Governorate, has been selected to represent the faculties of dentistry in Upper Egypt, as it is one of the public dental faculties in Upper Egypt. FDBSU offers a five-year dental program for undergraduate students. The first three academic years focus on pre-clinical courses (pre-clinical phase), while the last two levels (clinical phase) primarily focus on clinical courses and provide hands-on clinical experience with dental patients.

### Reliability and validity

Validity and reliability of the questionnaire had been evaluated using factor analysis and Cronbach’s Alpha test, as presented in Table 1. The overall DREEM tool reliability evaluated by Cronbach’s Alpha test was 0.94, indicating that the internal consistency was excellent for all questions, as they all were related to each other. Furthermore, exploratory factor analysis (EFA) demonstrated that the DREEM tool had an excellent factorability and can be divided into five subscales, as for Kaiser-Meyer- Olkin KMO value was 0.916. A significant difference (*P* < 0.000) was shown using Bartlett’s Test, and the total variance of DREEM questionnaire tool explained about 43.09% of students’ perception of their dental EE.The Kaiser-Meyer-Olkin (KMO) measure and Bartlett’s test results shared in validating the factorability of the DREEM tool. When applying these tests to DREEM, the KMO statistic evaluates the sampling adequacy for factor analysis, with values ideally above 0.5 indicating sufficient overlap among items to justify the analysis. Bartlett’s test assesses whether correlations between items differ significantly from an identity matrix, confirming that factor analysis is appropriate. These tests help confirm the construct validity and reliability of the DREEM questionnaire by demonstrating good factorability of its items in studying educational environments. This methodology has been used in various validation studies of the DREEM tool, such as in clinical and educational settings, to ensure robust psychometric properties. This aligns with the results of Serrano et al.,2020 [23], Gil et al.,2023 [29] and Armencia et al.,2024 [37].

Based on the findings of the current study, the null hypothesis was accepted as there was no statistically significant difference regarding the total DREEM score among students in FDBSU; however, it was partially rejected as two of the subscales presented statistically significant differences between students of different academic years.

Interpretation of demographic data in Table 2 revealed that 235 students participated in this study with a response rate of 47.5%. This aligns with the results of other dental schools in Saudi Arabia [25] and Pakistan [38]. In contrast, a higher response rate had been reported by Ahmad et al.,2015 [39], Kossioni et al.,2012 [40]. Similarities and differences in the literature regarding the response rate may be due to inconsistent timing of data collection, method of administration, and motivation to participate in the questionnaire. Despite the moderate response rate in our study, which may be attributed to fatigue of students as they often face multiple surveys throughout their academic year, the diversity of participants allowed for good representation of most students at FDBSU. The gender distribution in our results showed that participation of females (55.3%) was higher than that of males (44.7%), with no significant differences between them in their participation and total DREEM score. This is consistent with the results of Askari et al.,2018 [41] and Al Moaleem et al.,2020 [42] who reported comparable participation of both genders in their studies [41, 42]. This reflects that the results represented all students, rather than being biased toward one gender’s perspective.

The impact of the location of permanent residence on the results of the DREEM questionnaire is linked with various factors including socioeconomic status, resource availability, and the cultural context, all of which can positively or negatively affect the students’ perception of their EE. This complexity makes the interpretation of the differences in the location of permanent residence independent of other factors very difficult [13]. In our study most of the students in FDBSU are residents of Beni-Suef governorate (64.25%), while 36.75% of the students travel or move from other governorates to study in FDBSU. There was no significant difference in the total DREEM score concerning the permanent residence location of students Within the limitations of the available data, this may indicate adaptation of students from other governorates to traveling and/or accommodation in Beni-Suef. This may be due to the strategic geographic location of Beni-Suef, as it connects both the northern and southern regions of Egypt, as well as the eastern and western parts. It is located close to significant governorates, including Cairo and Giza. Also, the existence of major highways and railways facilitates the transportation of students from various governorates to FDBSU [43].

In recent years, there has been a notable influx of both Egyptian and international students transferring from conflict-affected universities in Sudan, Ukraine, and Russia to FDBSU. These factors have significantly contributed to the diversity among students at FDBU [44, 45]. International students, who represented 6.4% of the responses to the questionnaire, had a slightly lower total DREEM score than Egyptian students, with no significant difference between the two groups. This aligns with Dávidovics et al., 2024, who reported in a study conducted in the University of Pécs Medical School and the University of Szeged, that the total DREEM scores for both Hungarian and international dental and medical students indicated a “more positive than negative” perception of their educational environment [33]. The satisfaction of international students in our study and Dávidovics et al., 2024 may be attributed to similar security and political stability in both countries., especially given the unstable security situation caused by widespread regional conflicts in the Middle East. Additionally, the lower tuition fees for international students in Egypt, compared to other countries, contributed to their increased satisfaction with the EE at FDBSU [46].

### Overall DREEM score

The total DREEM score of all academic years at FDBSU, presented in Table 1, yielded a mean value of 128.59 ± 25.4 out of 200, indicating ‘a more positive than negative’ perception of students toward their EE. This finding aligns with the results of numerous dental schools worldwide (Table 5 Supp) [25, 31, 33, 47–51]. For instance, Ostapczuk et al.,2012 reported a total score of 122.95 ± 15.52 for a German dental school [21]. In Saudi Arabia, Al-Ahmari et al.,2022 noted in their systematic review that ten dental colleges had total scores ranging from 101 to 150, with only one study from Umm Al-Qura University scoring above 150, indicating an excellent perception of EE there [31]. The ‘more positive than negative’ perception highlights strengths in several aspects of EE at FDBSU, while acknowledging the presence of areas that require improvement.

### Cross-year comparison

Regarding different academic years, the mean DREEM scores for the five academic years in FDBSU presented in Table 2 were 127.06 ± 25.15 for the first level, 131.22 ± 25.71 for the second level, 123.65 ± 26.30 for the third level, 123.94 ± 25.06 for the fourth level, and 136.97 ± 23.81 for the fifth level. Despite some variations, statistical assessments revealed no significant differences across all five levels. This suggests that all students, regardless of the academic level, share comparable perceptions of the EE. These results align with Aldowsari et al.,2021 who reported that the DREEM scores were consistent across year groups or stages in the curriculum [52], unlike Alfakhry et al.,2023, who reported that the perception of 4th year students was significantly lower than the rest of the students in other academic years [28]. Absence of significant variation in our study and Aldowsari et al.,2021implies that the overall learning environment, in both institutions is steady during the students’ academic progression.

Assessment of the effect of students’ transition from the preclinical to the clinical phase was crucial in identifying the strengths and challenges associated with each stage. Our findings in Tables 2 and 3 indicated that the overall DREEM score for the preclinical phase was slightly higher than that of the clinical phase, although this difference was not statistically significant. Moreover, no significant differences were observed between the two phases across the five subscales of the questionnaire. Similar findings were reported by Aldowsari et al.,2021 [52], while Alfakhry et al.,2023 [28], Waqar et al., 2024 [53] and Sabbagh et al.,2020 [25] reported a higher score for the preclinical phase with a statistically significant difference between the two phases. This reflects the stability of the EE elements in FDBSU across the preclinical and clinical phases.

### Subscale analysis

Analysis of the DREEM subscales across different academic years, presented in Table 4, revealed significant differences across academic years in SPL and SAP, while the SPT, SPA and SSP showed insignificant differences across the years. Regarding SPL, the fifth academic level showed the highest SPL at (33.19 ± 4.51), while the fourth academic level had the lowest SPL (29.57 ± 5.52) among students in FDBSU. This contrasts with Doshi et al.,2014 [54] and Nisa et al.,2024 [53], who reported that the fifth-year students had the lowest learning perception compared to other years. High SPL in the fifth academic level in our study may be due to increased familiarity with the clinical aspect of their education. Gaining more hands-on experience often makes their learning feel more practical and motivating, which increases their academic confidence and satisfaction with their EE. In contrast, the fourth year, which marks the beginning of their clinical stage involving patient interactions, greater responsibilities, and clinical requirements, can lead to more stress. This may result in them feeling less positive about the EE at the fourth level.

Regarding SPT, there was no statistically significant difference in SPT across different academic years. This was consistent with Al Moaleem et al.,2020 [42] who reported that there was no statistically significant difference in SPT among students. In contrast, Doshi et al.,2014 [54], and Alraawi et al.,2020 [55] reported a significantly low SPT for students in the fourth academic level. The consistent SPT scores among participants in our study may be attributed to steady teaching quality, and uniform feedback and mentorship, making students tend to perceive their educators similarly, regardless of their academic level.

Students’ Academic Self-Perceptions (SAP) showed a significant difference among students. Fifth-level students had the highest SAP score (22.94 ± 4.53), while third-level students had the lowest SAP score (19.38 ± 4.86). This aligns with Vaughan et al.,2015 [56], who reported a higher SAP of students in the clinical phase, unlike Ahmad et al.,2015 [39], who reported that there was no statistically significant difference between seniors’ SAP and their perception when they were in the first academic level. The high SAP among the fifth-year students likely reflects their growing confidence in their abilities. This increase may be due to their mastery of skills, expanded knowledge, and effective adaptation to challenges, especially within the EE designed to promote their ongoing development.

SPA and SSP showed no significant difference among students at all academic years. This aligns with Ahmad et al.,2015 [39] and Sathyan et al.,2025 [57]. Conversely, Awad et al.,2025 [58] found that SPA was significantly lower in clinical students compared to preclinical students, while SSP levels remained stable across different phases. The consistency of SPA and SSP in our study is likely because these subscales are influenced by institutional culture, peer interactions, and the overall social climate, which do not vary greatly between academic years. In contrast, academic self-perception and perception of learning are more sensitive to curriculum challenges and responsibilities that change across different academic years.

A comparative analysis of the total scores across all subscales presented in Table 4 revealed that all subscales reflected a predominantly positive perception among students. The highest total subscale score was observed for SPL (30.57 ± 5.90), suggesting that educators at FDBSU are moving in the right direction, while the lowest score was for SSP (16.45 ± 5.2), indicating that students’ social self-perceptions were not too bad. This aligned with Ellawala et al.,2021 [59] and Ahmed et al.,2018 [60]. The correlation among the five subscales presented in Table 5, demonstrated a significant positive moderate correlation between the subscales. This indicated that the increase in one subscale will directly cause an increase in all subscales. This finding is consistent with that reported by Altawaty et al.,2020 [61], Singh et al.,2023 [62] and Bakhshialiabad et al.,2015 [63] who observed positive correlations among all subscales of the DREEM questionnaire. The most significant correlation was observed between SPA and both SAP (0.76) and SPL (0.72). This finding suggests that a supportive environment promotes enhanced engagement and motivation, leading to improved academic performance and greater overall satisfaction among students.

### Problematic items and educational implications

Interpretation of the individual item scores revealed the challenging items in the EE. Items that had low scores of less than 2 were considered problematic aspects in FDBSU. As demonstrated in Table 6, challenging items were observed in all subscales except for SAP, which was satisfactory with the absence of challenging items. This indicated that students had high confidence in their academic abilities. Our results aligned with multiple studies worldwide that reported the same aspects as frequently challenging items in medical and dental schools [58, 64–67]. Challenging items reflected predominance of factual learning that focuses mainly on memorizing and recalling facts, also teacher-centered learning that, despite its benefits in keeping classroom order and clear structure, may restrict student engagement and critical thinking development. The reported irritation of teachers by students may stem from this restrictive environment, where students’ roles are often passive [58]. Students expressed dissatisfaction with fatigue and stresses that negatively impact their enjoyment during their courses. Additionally, experience of boredom during the courses reflected weaknesses in teaching methods, content relevance, or the atmosphere that fail to stimulate active learning and participation.

The challenging aspects of the EE in FDBSU could be overcome by making courses more interactive and student-centered. This includes the incorporation of innovative teaching methods and active learning strategies, such as case-based discussions, problem-based learning, and frequent formative assessments that increase engagement and reduce boredom [62, 68]. Recently, research supported student-centered learning in which educators’ roles shift from knowledge transmitters to facilitators who encourage students to actively collaborate and take charge of their learning, thereby improving their academic performance and reducing tension within the classroom. Additionally, the incorporation of recent technology and artificial intelligence into education can positively impact and enrich learning experiences [69, 70]. Adopting these approaches into the EE can help balance enjoyment and stress, strengthen the relationships between educators and students, and foster deeper, more engaging learning experiences.

### Limitations

The limitation of the current cross-sectional study is that it was conducted in only one faculty of dentistry in Upper Egypt with potential response bias due to voluntary online participation, single-site generalizability, and self-report bias. Therefore, future research involving both public and private dental faculties across Egypt may provide a better understanding of the dental EE in Egypt. Additionally, conducting longitudinal studies to follow students throughout all academic years will offer deeper insights into their perceptions. Updating the DREEM questionnaire by adding a qualitative component will allow students to express their feedback more clearly. One of the limitations is that the lack of qualitative insight to explain why some items scored low as cross-sectional design cannot infer causality. Finally, evaluating the EE of interns and postgraduates could provide a more comprehensive understanding of EE in this region.

## Conclusion

As the first cross-sectional study evaluating the dental EE in Upper Egypt using the DREEM tool, we concluded that perceptions of students regarding the EE unveil equality that was positive across genders, nationalities, or preclinical and clinical phases. Moreover, it unveilsgood quality by giving everyone the tools and chances they require to realize their greatest potential. Tailored support might be required across different academic years in 2 subscales, SPL and SAP. With the presence of a strong correlation, it could be stated that enhancement in any of the elements will positively improve all aspects of the EE. Future curriculum enhancement should focus on balancing factual and conceptual learning, fostering student autonomy, and improving psychosocial support.

### Recommendations

Based on the results of the current study, we recommend the following:

1. Promote student-centered teaching and mentoring support
2. Integration of new teaching methods that increase students’ engagement in the classroom.
3. Incorporation of recent technology and artificial intelligence into dental education.
4. Improvement of academic supervision to provide continuous monitoring and support to students.
5. Continuous improvement of the atmosphere to create a more positive and motivating EE.
6. Students and staff professional development is highly recommended through faculty workshops.
7. Continuous evaluation and analysis of students’ feedback systems is also recommended.

## Supplementary Information

Supplementary Material 1.

## Data Availability

The datasets related to this study are available on reasonable request.

## Abbreviations

EE: Educational Environment
FDBSU: Faculty of Dentistry, Beni-Suef University
DREEM: Dundee Ready Education Environment Measure
SPL: Students’ Perception of Learning
SPT: Students’ Perceptions of Teachers
SAP: Students’ Academic Self-perceptions
SPA: Students’ Perceptions of Atmosphere
SSP: Students’ Social Self-perceptions
