# Supplementary material for: Evaluation of the dental educational environment in upper Egypt using the DREEM tool: a cross-sectional study
Source: BMC Med Educ. 2025 Dec 13;26:102. doi: 10.1186/s12909-025-08340-y (PMC12821931; doi:10.1186/s12909-025-08340-y)
Supplement: Supplementary file 1 — Supplementary Material 1. [file 12909_2025_8340_MOESM1_ESM.docx]

**The supplementary materials**

**Statistical analysis:**

Data was analyzed using SPSS version 22, with a significance level set at 0.05. Basic demographic data of the participants were treated as qualitative variables, while the categories of the dental educational environment were presented using frequency and percentage. A Z-test for proportions was applied to compare response rates across demographic variables. Independent t- tests and one-way ANOVA (F-test) were then used to examine differences in the total questionnaire score and each of the five subscales, with results expressed as mean ± standard deviation (x ± SD), according to demographic data, academic years, and academic phase. The reliability of the questionnaire was assessed using Cronbach’s alpha and exploratory factor analysis (EFA) with Principal Axis Factoring, setting a minimum factor loading of 0.4. The analysis included the Kaiser–Meyer–Olkin (KMO) measure of sampling adequacy, Bartlett’s test, communalities extraction, and eigenvalues greater than 1. Confirmatory factor analysis was subsequently conducted using AMOS software version 22 to confirm the five-factor structure. (figure 4) EFA was used to examine the underlying factor structure of the DREEM questionnaire in our study population to test internal consistency and factor structure in the local context. The association between dental educational environment categories and academic years was examined using the Monte Carlo test (2-sided). Finally, correlations among the five subscales of the dental EE questionnaire were evaluated using Pearson’s correlation coefficient. The assumption of normality for the questionnaire scores was verified using the Kolmogorov–Smirnov test.

**Table 1 Supp: The factorability and variability of the questionnaire**

| **KMO and Bartlett's Test** | | |
| --- | --- | --- |
| Kaiser-Meyer-Olkin Measure of Sampling Adequacy. | | 0.92 |
| Bartlett's Test of Sphericity | Approx. Chi-Square | 6110.25 |
|  | df | 1225 |
|  | Sig. | 0.000 |

-KMO and Bartlett's Test

Table 1 demonstrated that Kaiser-Meyer-Olkin KMO value was above 0.5. results from the Bartlett's test were significant indicating that the questionnaire can be divided into subscales or domains (factorability) with variability in participates’ responses.

**Table 2 Supp: The total variance explained by the questionnaire**

| Factor | Initial Eigen values | | | Communalities | Extraction Sums of Squared Loadings | | | Rotation Sums of Squared Loadings | | |
| --- | --- | --- | --- | --- | --- | --- | --- | --- | --- | --- |
|  | Total | % of Variance | Cumulative % | Extraction | Total | % of Variance | Cumulative % | Total | % of Variance | Cumulative % |
| 1 | 16.15 | 32.30 | 32.30 | .465 | 15.613 | 31.226 | 31.226 | 6.914 | 13.829 | 13.829 |
| 2 | 2.67 | 5.34 | 37.64 | .374 | 2.089 | 4.178 | 35.404 | 6.082 | 12.164 | 25.993 |
| 3 | 2.1 | 4.19 | 41.83 | .201 | 1.548 | 3.096 | 38.500 | 3.135 | 6.269 | 32.262 |
| 4 | 1.74 | 3.5 | 45.33 | .523 | 1.210 | 2.421 | 40.921 | 3.009 | 6.018 | 38.280 |
| 5 | 1.62 | 3.24 | 48.57 | .411 | 1.087 | 2.173 | 43.094 | 2.407 | 4.814 | **43.094** |
| 6 | 1.56 | 3.12 | 51.69 | .454 |  |  |  |  |  |  |
| 7 | 1.48 | 2.97 | 54.66 | .419 |  |  |  |  |  |  |
| 8 | 1.22 | 2.45 | 57.1 | .359 |  |  |  |  |  |  |
| 9 | 1.1 | 2.24 | 59.34 | .346 |  |  |  |  |  |  |
| 10 | 1.05 | 2.11 | 61.45 | .544 |  |  |  |  |  |  |
| 11 | 1.01 | 2.02 | 63.46 | .357 |  |  |  |  |  |  |
| 12 | .99 | 1.99 | 65.45 | .199 |  |  |  |  |  |  |
| 13 | .96 | 1.92 | 67.37 | .453 |  |  |  |  |  |  |
| 14 | .90 | 1.80 | 69.17 | .416 |  |  |  |  |  |  |
| 15 | .86 | 1.72 | 70.88 | .380 |  |  |  |  |  |  |
| 16 | .84 | 1.69 | 72.57 | .476 |  |  |  |  |  |  |
| 17 | .81 | 1.61 | 74.18 | .466 |  |  |  |  |  |  |
| 18 | .73 | 1.46 | 75.64 | .419 |  |  |  |  |  |  |
| 19 | .71 | 1.40 | 77.04 | .400 |  |  |  |  |  |  |
| 20 | .69 | 1.38 | 78.41 | .408 |  |  |  |  |  |  |
| 21 | .65 | 1.31 | 79.72 | .497 |  |  |  |  |  |  |
| 22 | .62 | 1.25 | 80.97 | .410 |  |  |  |  |  |  |
| 23 | .57 | 1.13 | 82.10 | .324 |  |  |  |  |  |  |
| 24 | .56 | 1.13 | 83.23 | .342 |  |  |  |  |  |  |
| 25 | .55 | 1.09 | 84.32 | .428 |  |  |  |  |  |  |
| 26 | .51 | 1.01 | 85.33 | .598 |  |  |  |  |  |  |
| 27 | .47 | .93 | 86.26 | .400 |  |  |  |  |  |  |
| 28 | .46 | .92 | 87.18 | .534 |  |  |  |  |  |  |
| 29 | .44 | .88 | 88.06 | .268 |  |  |  |  |  |  |
| 30 | .43 | .86 | 88.91 | .600 |  |  |  |  |  |  |
| 31 | .41 | .81 | 89.72 | .389 |  |  |  |  |  |  |
| 32 | .4 | .79 | 90.51 | .394 |  |  |  |  |  |  |
| 33 | .39 | .78 | 91.29 | .416 |  |  |  |  |  |  |
| 34 | .36 | .73 | 92.02 | .154 |  |  |  |  |  |  |
| 35 | .35 | .71 | 92.73 | .421 |  |  |  |  |  |  |
| 36 | .35 | .69 | 93.41 | .425 |  |  |  |  |  |  |
| 37 | .33 | .65 | 94.06 | .503 |  |  |  |  |  |  |
| 38 | .31 | .62 | 94.69 | .508 |  |  |  |  |  |  |
| 39 | .31 | .61 | 95.30 | .460 |  |  |  |  |  |  |
| 40 | .29 | .58 | 95.88 | .534 |  |  |  |  |  |  |
| 41 | .28 | .56 | 96.44 | .536 |  |  |  |  |  |  |
| 42 | .26 | .52 | 96.95 | .707 |  |  |  |  |  |  |
| 43 | .26 | .51 | 97.47 | .522 |  |  |  |  |  |  |
| 44 | .22 | .44 | 97.91 | .465 |  |  |  |  |  |  |
| 45 | .21 | .42 | 98.33 | .342 |  |  |  |  |  |  |
| 46 | .2 | .39 | 98.72 | .422 |  |  |  |  |  |  |
| 47 | .19 | .38 | 99.10 | .545 |  |  |  |  |  |  |
| 48 | .17 | .35 | 99.44 | .767 |  |  |  |  |  |  |
| 49 | .17 | .33 | 99.77 | .353 |  |  |  |  |  |  |
| 50 | .11 | .23 | 100.000 | .211 |  |  |  |  |  |  |
| Factor analysis Extraction Method: Principal Axis Factoring. | | | | | | | | | | |

Table 2, using principal axis factoring, reported that the five subscales of the questionnaire explained 43.094% of the dental educational environment. Each question showed a strong correlation with the total questionnaire score. None of the questions could be excluded as the communalities extraction values were above 0.1.

**Table 3 Supp: Factor loading by Amos program**

| Subscale | Items | |  | Factor analysis (factor loading) | | | | |
| --- | --- | --- | --- | --- | --- | --- | --- | --- |
|  |  |  |  | **Factor 1** | **Factor 2** | **Factor3** | **Factor4** | **Factor5** |
| Total  DREEM score | |  | |  | | | | |
| Students’ Perception of Learning (SPL) | Q1 |  | | 0.7 |  |  |  |  |
|  | Q2 |  | | 0.64 |  |  |  |  |
|  | Q3 |  | | 0.44 |  |  |  |  |
|  | Q4 |  | | 0.71 |  |  |  |  |
|  | Q5 |  | | 0.52 |  |  |  |  |
|  | Q6 |  | | 0.69 |  |  |  |  |
|  | Q7 |  | | 0.59 |  |  |  |  |
|  | Q8 |  | | -0.55 |  |  |  |  |
|  | Q9 |  | | 0.59 |  |  |  |  |
|  | Q10 |  | | 0.75 |  |  |  |  |
|  | Q11 |  | | 0.61 |  |  |  |  |
|  | Q12 |  | | -0.17 |  |  |  |  |
| Students’ Perceptions of Teachers (SPT) | Q13 |  | |  | 0.67 |  |  |  |
|  | Q14 |  | |  | 0.64 |  |  |  |
|  | Q15 |  | |  | 0.44 |  |  |  |
|  | Q16 |  | |  | 0.49 |  |  |  |
|  | Q17 |  | |  | 0.7 |  |  |  |
|  | Q18 |  | |  | 0.7 |  |  |  |
|  | Q19 |  | |  | 0.65 |  |  |  |
|  | Q20 |  | |  | 0.64 |  |  |  |
|  | Q21 |  | |  | 0.39 |  |  |  |
|  | Q22 |  | |  | 0.64 |  |  |  |
|  | Q23 |  | |  | -0.30 |  |  |  |
| Students’ Academic self-perceptions (SAP) | Q24 |  | |  |  | 0.55 |  |  |
|  | Q25 |  | |  |  | 0.66 |  |  |
|  | Q26 |  | |  |  | 0.78 |  |  |
|  | Q27 |  | |  |  | 0.66 |  |  |
|  | Q28 |  | |  |  | 0.7 |  |  |
|  | Q29 |  | |  |  | 0.51 |  |  |
|  | Q30 |  | |  |  | 0.79 |  |  |
|  | Q31 |  | |  |  | 0.59 |  |  |
| Students’ Perceptions of Atmosphere (SPA) | Q32 |  | |  |  |  | 0.58 |  |
|  | Q33 |  | |  |  |  | 0.6 |  |
|  | Q34 |  | |  |  |  | 0.16 |  |
|  | Q35 |  | |  |  |  | 0.65 |  |
|  | Q36 |  | |  |  |  | 0.65 |  |
|  | Q37 |  | |  |  |  | 0.68 |  |
|  | Q38 |  | |  |  |  | 0.72 |  |
|  | Q39 |  | |  |  |  | 0.41 |  |
|  | Q40 |  | |  |  |  | 0.66 |  |
|  | Q41 |  | |  |  |  | 0.66 |  |
|  | Q42 |  | |  |  |  | 0.8 |  |
|  | Q43 |  | |  |  |  | 0.73 |  |
| Students’ Social self-perceptions (SSP) | Q44 |  | |  |  |  |  | 0.67 |
|  | Q45 |  | |  |  |  |  | 0.48 |
|  | Q46 |  | |  |  |  |  | 0.65 |
|  | Q47 |  | |  |  |  |  | 0.45 |
|  | Q48 |  | |  |  |  |  | 0.65 |
|  | Q49 |  | |  |  |  |  | 0.52 |
|  | Q50 |  | |  |  |  |  | 0.32 |

-AMOS program version 22

Table 3 shows the correlation between each question and the subscale.

**Table 4 Supp: The dental educational environment categories in different academic ~~levels~~ years**

| **Dental educational environment category** | **Frequency** | **Percent** | **Academic year of education** | | | | | **Monte Carlo Sig.**  **(2-sided)** | |
| --- | --- | --- | --- | --- | --- | --- | --- | --- | --- |
|  |  |  | **First**  year | **Second**  year | **Third**  year | **Fourth**  year | **Fifth**  Year | **X2** | **P** |
| **Very poor EE** | 1 | 0.4 | 0 | 0 | 1 | 0 | 0 | 13.318 | 0.310b |
|  |  |  | 0% | 0% | 100.0% | 0% | 0% |  |  |
| **plenty of problems EE** | 34 | 14.5 | 11 | 7 | 3 | 11 | 2 |  |  |
|  |  |  | 32.4% | 20.6% | 8.8% | 32.4% | 5.9% |  |  |
| **more positive than negative EE** | 155 | 66.0 | 37 | 39 | 18 | 34 | 27 |  |  |
|  |  |  | 23.9% | 25.2% | 11.6% | 21.9% | 17.4% |  |  |
| **excellent EE** | 45 | 19.1 | 14 | 14 | 4 | 6 | 7 |  |  |
|  |  |  | 31.1% | 31.1% | 8.9% | 13.3% | 15.6% |  |  |

-Monte Carlo Sig. (2-sided)

Based on the total DREEM score categories of the dental EE have been ~~illustrated in (Figure 4),~~ presented in Table 4, showing that most of the students (66%) perceived the EE as more positive than negative, while 19.1% considered it as excellent EE. 14.5% of students perceived that EE has plenty of problems, while 0.4% perceived it as very poor EE. Table 5 also demonstrated that there was no association between dental EE category and academic level of education, as the association was insignificant (*P*= 0.310).

**Table 5 Supp: a summary table comparing DREEM mean in the current study with other countries in previous studies**

|  | Researcher(s)/ publication year | Overall DREEM score | Interpretation |
| --- | --- | --- | --- |
| Egypt | Our results | 128.59±25.4 | more positive than negative EE |
| Saudi Arabia | Arora et al.,2021  [20] | 130.87±32.73 | more positive than negative EE |
| Germany | Ostapczuk et al.,2012 [21] | 122.95 ± 15.52 | more positive than negative EE |
| Saudi Arabia | Sabbagh et al.,2020 [25] | 125 | more positive than negative EE |
| Syria | Alfakhry et al.,2023  [28] | 108.8 § 31.5 | more positive than negative EE |
| Saudi Arabia | Al-Ahmari et al.,2022 [32] | 89.2-170.9 | A systematic review includes all categories |
| Hungary | Dávidovics et al., 2024 [34] | 118.1 for international students,  122.6 for Hungarian students | more positive than negative EE |
| India | Doshi et al.,2014 [49] | 125.24 + 21.10 | more positive than negative EE |
| India | Saha et al.,2024 [50] | 117 to 138 | more positive than negative EE |
| New Zealand | Page et al.,2011  [51] | Bachelor of dental surgery 141.4 (15.9)  Bachelor of oral health 142.1 (16.2) | more positive than negative EE |
| Turkey and  Pakistan | Zafar et al.,2020 [52] | 119.34 Â±24.26 for the Pakistan college  113.85 Â± 16.13 for the Turkish college | more positive than negative EE |
